# The Treatment of Extra-Uterine Pregnancy

**Published:** 1907-07

**Authors:** Aime Paul Heineck

**Affiliations:** Chicago, Ill., Surgeon to the Samaritan and Cook County Hospital; Professor of Surgery, Dearborn Medical College; Adjunct Professor of Surgery, College of Physicians and Surgeons, University of Illinois


TEXAS MEDICAL JOURNAL.

Established July, 1885.

F. E. DANIEL, M. D., - - - - Editor, Publisher and Proprietor.

PUBLISHED MONTHLY.—SUBSCRIPTION $1.00 A YEAR.

VOL. XXIII. AUSTIN, JULY, 1907. No. 1.

The publisher is not responsible for the views of contributors.

For Texas Medical Journal.

### The Treatment of Extra-Uterine Pregnancy.

BY AIME PAUL HEINECK, CHICAGO, ILL.,

Surgeon to the Samaritan and Cook County Hospital; Professor of Surgery, Dearborn Medical College; Adjunct Professor of Surgery, College of Physicians and Surgeons, University of Illinois.

Extra-uterine pregnancy is of far more frequent occurrence than the current literature of the subject would indicate. The diagnosis of this condition is often a matter of great difficulty. Hence, the failure to recognize, or rather to diagnose this condition, is largely responsible for the morbidity and the mortality which so frequently accompanies extra-uterine gestation.

A knowledge of the possible termination is essential before there can be a rational discussion of the methods of treatment:

1. Gestation may go to term and a viable child be delivered through channels created by the surgeon. This mode of termination is exceptional. Even when it does occur the child may live but a few days. It is frequently the subject of malformations. These malformations are due to the unnatural conditions of existence under which the extra-uterine child develops. The operation necessary for their delivery may determine the death of the mother or child, and frequently of both. Both the ovarian and tubal varieties of pregnancy may be terminated by the surgical delivery of a viable child. In the absence of surgical intervention, an extra-uterine pregnancy always terminates fatally for the child, and very frequently so for the mother.

2. Gestation may go to term, at which time the fetus may die and remain undelivered, persisting in the maternal organism as a cyst, a fetal cyst.

3. The fetus may die at any time previous to term or at term.

If the gestation be advanced in development, the ovum will not be absorbed.

In all cases, where the fetus is retained, the life of the mother is menaced on account of the degenerative and other changes that may take place in the fetus and in its envelopes. The life of a woman who carries an encysted fetus is in continuous peril.

Degenerative changes may take the form of: (a) Putrefaction; (b) of infection; (c) the fetus may persist in a cartilaginous state; (d) it may be converted into a friable fatty soap-like substance and then be known as an adipocere; or, (e) it may be changed into a lithopedion; or, (f) a connective tissue membrane may form a capsule encysting the fetus.

Fetal cysts may cause (1) mechanical interference with the development of a uterine pregnancy; may complicate the delivery of a uterine fetus; (2) may be a source of mechanical irritation to contiguous organs, producing symptoms of dysuria, rectal and vesical tenesmus, and (3) by mechanical compression of neighboring organs may cause symptoms of intestinal obstruction, urinary retention, as well as various displacements of the uterus.

On the death of the fetus, the liquor amnii is absorbed, no more is secreted, the cyst shrinks and the product of conception remains in the maternal organism.

Full-term extra-uterine fetuses are rarities. However, they have been found in the abdominal cavity at necropsies, and also at the operating table.

On death of the fetus the gestation sac should be regarded and treated as a cyst, a fetal cyst. Fetal cysts frequently become adherent to neighboring organs and to neighboring tissues, and by ulcerating into or through them may discharge their contents partially or completely into the bowel, the vagina, urinary bladder, into the uterus, etc. Many cases are reported in which the fetal cysts opened into the rectum and discharged their contents per ano. They have been known to become adherent to, and by means of an ulcerative inflammation to eventually discharge their contents through the abdominal walls.

There may be more than one channel of elimination, as in Fenwick's[1] case, in which the gestation sac, of several years' duration, contained a macerated fetus and had formed two fistulae, one opening into the bowel, and the other into the bladder. Urbain[2]

---

I. E. H. Fenwick, British Med. Journal, 1904, Vol. II, p. 1739.

2. Urbain, Bull de la Societe de Gynecologie et d'Obstetrique, Bruxelles, 1900.

reports a case in which a left tubal gestation sac was found to have ruptured into a cyst of the right ovary.

In the tabulated cases reported by Parry[3], Mattei and Peuch, it is seen that 89 ruptured through the abdominal walls. Of these, 15 died. There were 164 cases of rupture into the intestinal canal with 47 deaths; 42 ruptured into the vagina, with 12 deaths, and 34 through the bladder, with 10 deaths.

Rupture is a frequent termination of all forms of extra-uterine gestation. This accident may occur either before or after the death of the fetus. Primary rupture most frequently takes place between the third and tenth week of gestation. Rupture of tubal pregnancies almost always take place before the end of the fourth month. Both the primary and secondary gestation sacs are liable to rupture. Hemorrhage always accompanies rupture. This hemorrhage may be slight; may be massive; may be single; may be repeated. Rupture is either extra-tubal, intra-tubal or intra-mural, or may show the characteristics of two or more of these varieties. If the tension in the gestation sac is not relieved by rupture in one direction, there is great probability of secondary rupture occurring in another direction.

Cessation of gestation is frequently caused by rupture. It is asserted that the loss of the amniotic fluid invariably ends gestation. The literature of the subject records only one case in which, at the time of operation, no amniotic sac could be discovered. Absorption of the ovum will be quicker when the rupture occurs early. Absorption will not occur when the ovum dies at an advanced period of its development. The retention of these ova is a menace to the mother and may cause her death, owing to necrosis of the fetal sac and of the vessels supplying it. It may cause prolonged illness and, eventually, death, owing to ulceration of the sac into contiguous organs. If the death of the ovum does not follow the rupture of the gestation sac into the peritoneal cavity, the pregnancy is continued as a tubo-peritoneal or peritoneal pregnancy.

A tubal sac may rupture between the folds of the broad ligament. If, under these circumstances, gestation continues, it is designated by some authors an intra-ligamentary pregnancy. Kustaer, however, in a series of 107 operations for ectopic gestation, never once found either an intra-ligamentary or a tubo-abdominal pregnancy. The primary rupture may be between the folds of the broad ligament and the secondary rupture take place into the peritoneal cavity. An intra-ligamentary pregnancy in de-

3. Parry, Extra-Uterine Pregnancy, a monograph. Philadelphia.

veloping may lift up the peritoneum from the anterior abdominal wall as it ascends, and thus become pro-peritoneal, or it may dissect up the peritoneum from the sacrum and thus become retroperitoneal.

If the abdominal opening be occluded at the time of an intratubal rupture, an hemato-salphinx results from the accumulation of blood in the cavity of the tube. Again, the ovum may continue to develop in the tube and a secondary rupture may occur, either in the peritoneal cavity or between the folds of the broad ligament, or in both. The hemorrhage may carry the ovum out of the tube. This migration of the ovum into the peritoneal cavity is known as a tubal abortion.

The most general and most dangerous result of a rupture is the hemorrhage which usually requires immediate surgical interference to save life. This hemorrhage is to be dreaded, for, at times, it is appalling.

Rupture may be due to hemorrhage:

(a) Between the chorion and tubal mucous membrane.

(b) To repeated small hemorrhages from the tubo-chorionic vessels into the gestation sac, causing overdistension of the latter, and consequent rupture.

(c) To the absence or feeble development of the decidua reflexa.

(d) To the cyst wall being thinned and weakened by the chorionic villi.

(e) To traumatism, even a very slight one may cause rupture.

It is now generally agreed that the trophoblasts are the active agents of early rupture, while mechanical causes, as undue violence in making examinations, etc., produce the late ruptures. These trophoblasts exert an erosive effect destroying the vascular tissues, as well as the muscular tissues.

It is well to bear in mind that the most severe hemorrhages may occur from very small orifices. The rupture itself varies in form. In size it may be punctiform, a large tear, or even an all but complete rending of the tube. These hemorrhages, be they large or small, are the most alarming accompaniment of tubal or of ovarian pregnancies. The amount of blood discharged bears no definite relation to the extent of the tubal or ovarian tear.

Rupture of ovarian or tubal gestation sacs usually occur before the third month. Attending rupture of an extra-uterine sac, there may be one severe and even fatal intra-peritoneal hemorrhage or there may be recurring hemorrhages, causing maternal death. The danger of recurring hemorrhages is one of the perils that attend the expectant treatment of this condition. Whether the hemorrhage be peritoneal, encysted or diffuse, and whether produced by a ruptured tubal or a ruptured ovarian sac, the prognosis is always grave. The extent, duration and continuousness of the hemorrhage and not its source, determine the final outcome. Retro-uterine hematoceles are usually caused by extra-uterine pregnancies, as is shown by the fact that frequently chorionic villi, and at times even an embryo may be demonstrated in them.

In all cases of extra-uterine pregnancy, hemorrhages, internal or external, occur at some period of their existence. A pelvic hematoma will result if the extra-tubal rupture occurs between the folds of the broad ligament, from the accumulation of blood separating the folds. This pelvic hematoma is usually unilateral, may be bilateral, becoming so by the passage of blood in front of or posteriorily to the uterus from the side of the pregnancy to the opposite side.

The migration of the ovum into the abdominal cavity is the definition given by Bland Sutton of tubal abortion. A tubal abortion may lead to an hematoma-salpinx. The blood may, after passing through the ostium abdominale, collect in the Douglas cul-de-sac, forming a retro-uterine hematocele, or may produce a condition of hemaperitoneum.

Tubal abortion may be caused:

1. By separation of the placenta from tubal contractions.
2. From hemorrhages taking place into the placental tissue.
3. From repeated small hemorrhages detaching the ovum.
4. From hemorrhage in the muscular fibres of the pregnant oviduct.
5. From intra-tubal hemorrhages, carrying the ovum out of the tube.

Death of the ovum, atrophy of the chorionic villi and consequent detachment of the ovum may be caused by an intra-ovulary hemorrhage.

The trophoblasts are the most frequent determining factors of abortion and rupture in the early period of extra-uterine gestation. The local, destructive and erosive action of these ectoblastic cells is exerted upon the maternal muscular tissues as well as upon the maternal vascular tissues at the point of implantation of the ovum. Tubal abortion usually occurs within the first month, although it has been reported as occurring as late as the ninth month (Martin). The union of the chorionic villi and tubal mucosa is not very intimate during the early periods of ectopic gestation, neither is the tube greatly distended, and the tubal ostium abdominale is patent. Under these circumstances, the muscular layers can furnish contractions sufficiently powerful to force the ovum and extravasated blood out of the tubal lumen into the abdominal cavity.

I have not the slightest hesitancy in advancing what I firmly believe to be the only safe and scientific method of treating ectopic gestation. I hold that this condition is a disease, a very dangerous disease, and that the ectopic-gestation sac should be regarded as a neoplasm, malignant at that. The extra-uterine ovum should not be treated as a pregnancy, unless the mother be extremely desirous of having a child, but as a parasitic or malignant growth. Surgery, therefore, offers the only reliable method of treatment. The surgeon's skill and science should be invoked on a diagnosis or even on a probable diagnosis of an ectopic pregnancy.

Three questions, however, must be decided before operation:

1. Shall the mother be abandoned to nature?
2. Will she live if operated?
3. Is there less danger in opening the abdominal cavity than that which may result from rupture of the gestation sac or from continuance of the pregnancy?

It must be remembered than an extra-uterine pregnancy always terminates fatally to the child, and very frequently to the mother unless relieved by the surgeon's art. The expectant plan of treatment is only too obviously dangerous. Of the 265 cases collected by Martin only 36.9 per cent recovered under the expectant treatment, while 76.7 per cent recovered under operative treatment. In another series of 515 cases Cestan says that internal hemorrhage due to tubal rupture is responsible for a maternal mortality of 83.1 per cent. A percentage of 84.4 of maternal recoveries after operation is shown by Choyan's.

Anesthesia, hemorrhage, shock and sepsis are the only dangers incident to operative interference in ectopic pregnancy. The first is but minimal, and the second can be minimized too, while shock can be greatly lessened by rapidity in operating. If careful aseptic and antiseptic precautions are observed, as they should be in all major operations, the dangers of sepsis can be almost eliminated.

McDonald says: "Time forbids entering into the discussion of the injection of narcotics into the gestation sac to induce death of the fetus, as well as that of the application of electricity in the treatment of extra-uterine pregnancy. These plans have been so repeatedly discussed and so universally condemned that nothing is to be gained by further consideration."

In the absence of urgent symptoms, we have, nevertheless, an operation of necessity. In case of rupture, whether hemorrhage has already occurred or is occurring, we have an emergency operation. It is not possible to see through the abdominal wall to determine whether the hemorrhage has ceased or is continuous. This can be done only by opening the abdomen. Even in the absence of urgent symptoms, the operation must not be delayed, because, as long as the fetus lives, the placenta keeps increasing in size and vascularity, thus vastly augmenting the dangers of operation.

C. Fenger said: "The early operation is technically no more difficult than the extirpation of the normal non-adherent uterine appendages. The operation toward the end of pregnancy, by which we mean the total removal of the ovum and of its contents, is always formidable, and often technically an absolute impossibility."

The co-existence of an intra-uterine pregnancy is no contra-indication for operation. The ectopic ovum must be removed; the uterine ovum must not be molested. It is frequently a very difficult matter to make a diagnosis of ectopic pregnancy. However, the conditions which simulate it require surgical interference and are only amenable to surgical measures.

The vaginal route is to be employed only in cases of pelvic abscess; that is, only in cases in which an ectopic fetal sac or a pelvic hematocele or a pelvic hematoma have undergone suppuration. Still, while the vaginal route is becoming deservedly more and more unpopular, it should not be entirely abandoned. Many surgeons employ both routes in cases in which either the one or the other proves insufficient. I prefer the abdominal route for the following reasons:

1. It enables the operator to attend to co-existing pathological conditions at the same time that he removes the gestation sac.

2. It enables one to secure a more complete and more careful hemostasis, the operative field being under much better control.

3. Hemorrhage can be arrested with greater rapidity; celerity is an important factor in operations involving the opening of the abdominal cavity.

4. The extent of damage can be better judged; a more direct examination can be made, and hence a more accurate diagnosis.

5. A more conservative ablation of organs can be made. There never should be any needless sacrifice of important organs, i. e., tubes and ovaries.

6. It is possible to get in contact with the exact conditions and also to remove more completely the fetal sac and its contents.

7. The sense of sight as well as that of touch can be utilized.

8. If a diagnostic mistake has been made, you have ready access to the conditions which stimulate ectopic pregnancy.

9. This is an abdominal condition, and to meet it a laparotomy is more likely to be successful than a vaginal section. At times the ovum is inaccessible by the vaginal route. It may ascend into the abdominal cavity.

If a pyosalpinx, hydrosalpinx, a co-existing tubal pregnancy of opposite tube, a benign or a malignant neoplasm be found in the opposite tube, remove it, of course. If the pathological conditions existing in the non-pregnant oviduct or ovary require their removal, the presence of ectopic pregnancy does not contraindicate it.

Many authorities express the opinion that extra-uterine pregnancy shows a tendency to recur, and, therefore, advise, as a prophylactic measure, the removal of the unaffected adnexae. I, however, am convinced that this procedure as a general rule should be energetically condemned. Normal uterine pregnancies have occurred after the removal of ectopic gestation sacs. Kynoch, Kokman and Orthman have reported cases of uterine pregnancies taking place in the interval between the occurrence of two extra-uterine pregnancies.

The hemorrhage is the one great difficulty to overcome in operating for extra-uterine pregnancy. The separation of adhesions and especially the removal of the placenta, are the causes of the terrific hemorrhages. Even a very slight "interference" with the placenta may cause a frightful hemorrhage. Hemorrhage may occur either at the time of operation or afterwards. The greatest danger from hemorrhage is when the fetus is still alive at time of operation, or if only recently dead. The following causes are advanced for the hemorrhage attending the separation of the placenta of an extra-uterine ovum:

1. The condition of the placenta which is still functionally active up to the moment of separating the fetus from it, if the fetus be alive.

2. The abnormal characteristics of the placenta itself.

3. The special and ectopic position of the placenta in each individual case.

4. The vascularity of the cyst wall.

5. The non-contractile basis upon which the placenta is located.

When the fetus has been dead for some time, the placenta can usually be removed without danger of hemorrhage. If the placenta has continued to develop in the tube, there is usually but slight complexity to the operation. The placenta has been found adherent in every possible way to every possible organ; to the parietal peritoneum, to the omentum, to the intestines, to the bladder and other intra-abdominal organs.

### OPERATION PROPER.

The patient should be catheterized immediately before being brought to the operating table. This enables the operator to avoid incising a distended bladder and removes a confusing element from the field of operation. The incision should be made to one side of the median line. The side selected will be determined by the vaginal findings at time of examination. A firmer cicatrix is thought to be more probable from a side incision. A side incision is better adapted to the method I employ in closing the abdominal wall. Avoid cutting the epigastric vessels and the urachus. The Trendelenberg position should be used. The patient should be placed gradually in this position, not suddenly. She should be just restored as gradually to a horizontal position after the operation. A thorough examination of the opposite tube and ovary should be made for evidences of a former or co-existant pregnancy or disease.

No needless sacrifice of tissues or organs is called for. If the opposite tube and ovary are unaffected, they should be unmolested.

Normal saline solution must not be given through any channel before the bleeding has been controlled or the bleeding vessels have been secured. When the bleeding points have been controlled, its use is of the utmost value. Before control of the bleeding has been effected, the normal salt solution adds to the difficulty by increasing the blood pressure and the resulting liability to dislodgment of the internal thrombi can be the cause of recurrence of the hemorrhage. If used before hemostasis has been secured, it will in most cases increase the blood flow. The operator should not close the abdomen until he is absolutely certain of his hemostasis. There must not be a suspicion or any misgiving as to this most important step in the operation.

Avoid denuding of peritoneal surfaces. Denuded surfaces offer avenues for the entrance of infection. Peritonization, or the covering of denuded surfaces with peritoneum, lessens the formation of adhesions. It also aids in checking the hemorrhage and forms a barrier to the advance of inflammatory processes.

I employ a peculiar method of suturing. I use an intra-dermic stitch (a subcuticular stitch), which is continuous and which does not penetrate the upper layers of the skin. The silkworm gut stitches are tied over a piece of gauze, extending from their point of entrance to their point of exit, and covering the line of incision.

The silkworm gut sutures are figure-of-eight sutures introduced after the introduction of the peritoneal sutures. The upper loop of the silkworm gut stitch includes both skin and subcutaneous tissues, the lower loop includes the sheath and the muscular fibers of the rectus. The peritoneal stitch includes peritoneum, properitoneal fat and transversalis fascia. It is so introduced as to evert the edges of the peritoneum, and the loop of the stitch does not appear in the peritoneal cavity. The fascial stitch of catgut restores the continuity of the sheath of the rectus. Voluminous gauze dressing is used, but no dusting powder. Zinc oxide adhesive plaster, because of its aseptic qualities, adhesiveness, and non-irritating properties, is used to hold the dressings in place.

Perineal straps passed around each thigh prevent the abdominal binder and of the dressings which cover the abdominal wound.

The greatest difficulty that arises in operating for ectopic pregnancy will be found in those cases in which the entire ovum can not be removed. In such cases, the principal difficulty lies in the removal of the placenta. The presence of dense adhesions of the cyst wall and the vascularity of the cyst wall itself are also difficulties which must not be ignored. The following methods have been employed:

1. The fetus, the umbilical cord and amniotic fluid have been removed, all else left in situ and the abdominal wall closed. This is an extremely risky measure and is not to be recommended. Some successes, however, have attended the employment of this method. The placenta, in such cases as are successful, is gradually and painlessly absorbed. It is bottled up, as it were, with no avenue of exit.

2. The amniotic fluid is evacuated, the umbilical cord is ligated close to its placental insertion. The fetus is removed and more or less of the sac is resected. The remainder is sutured to the margins of the wound in the abdominal wall. Then, for the first dressing, the cavity is tightly packed with gauze. After the first dressing, gauze is used daily for drainage purposes. This draining of the cavity and leaving the placenta and sac to be spontaneously expelled is the procedure most frequently employed. The sac must be reduced to its minimum before exteriorizing it.

3. The placenta is removed in part after removal of the fetus; that is, so much of it as can be separated easily and safely; the rest is left for spontaneous absorption.

4. The fetus is removed but the placenta left in situ. After an interval of time, when it is judged that the blood supply has been spontaneously cut off, the placenta is shelled out.

5. The ovum is removed in its entirety at one operation. This is the ideal measure. It should be attempted in all cases in which the enucleability of the entire ovum appears possible. Total extirpation of the ovum is the operation of election. It is a complete operation.

6. Placenta, gestation sac, together with the neighboring organs, uterus and ovaries, are removed at once, if the hemorrhage can not be arrested otherwise. This is the method employed in those cases which call for supra or total hysterectomy. These are cases in which the hemorrhage can not be stopped by any other procedure.

7. Vineberg, believing that leaving the placenta in situ increased the mortality sixfold, in a difficult case used the following original method:

The vessels of the unaffected side being first ligated (the uterine and ovarian vessels), he sectioned the uterus across at the level of the os internum, making no attempt to separate it from the placenta. The uterine artery on the diseased side was next ligated, and the operation finished by the removal of the uterus and the whole ectopic mass that was adherent to it. This operation is a partial hysterectomy.

8. Preliminary ligature of the uterine and ovarian arteries on the side which furnishes the blood supply to the placenta.

Martin noted that the tying of the uterine and ovarian arteries on the affected side controlled the hemorrhage to a remarkable extent. Though accessory vessels from the omentum mesentery, intestine or abdominal wall may contribute to the blood supply of the placenta; nevertheless, the main source of blood supply of the placenta are the enlarged branches of the ovarian and uterine arteries of the affected side.

The complete removal of the fetal sac and of the placenta is unquestionably the ideal procedure if consistent with the safety of the mother. Theoretically, the complete ablation of the ovum is the only perfect, the only complete, operation. It markedly shortens the patient's convalescence; eliminates the possible danger of sepsis that is liable to occur during the slow and tedious elimination of the placenta; does away with all the subsequent dressings of the case, and impairs to a less extent the integrity of the abdominal wall. In practice, it is not always possible.

In the case in which I feared to risk distrubing the placenta the sac was incised, the fetus and the other intraovulary contents were removed, and the umbilical cord was ligated close to its placental implantation. A portion of the sac wall was resected and the edges of the remaining portion were sewed to the abdominal wall. This method shuts off the general peritoneal cavity, exteriorizes the sac, as it were. A large extra-peritoneal pouch is the result, and this is packed with strips of aseptic gauze. Many surgeons use iodoform gauze. I have abandoned the use of iodoform gauze on account of some cases of intoxication attending its use which I have had. I now employ plain aseptic gauze instead. An effort is made to keep the sac aseptic until the placenta has sloughed wholly out of the wound. It takes from twenty to fifty days for the placenta to be eliminated in such cases.

Boissard reports two cases in which the placenta was treated by this method. In one, a case of a dead fetus, it took twenty-six days for the placenta to be completely eliminated. In the other, that of a living child, it took forty-five days. A vaginal drain was used in some cases in additional to the abdominal drain. The first strips of gauze that we insert in the fetal sac are made to serve the office of a compress or a tampon. They are used to check the bleeding. After the first dressings, the gauze strips are used more for drainage than for anything else. The compresses which have been used to protect the general peritoneal cavity are removed just immediately after the fetal cyst has been sewed to the abdominal wall or immediately before the latter step, varying according to the exigencies of the case. All communication between the cyst cavity and the peritoneal cavity is closed by this method of sewing the sac to the abdominal wall. I use No. 3 catgut for sewing the sac wall to the abdominal wall. Irrigation of this pouch with an astringent solution is employed by some clinicians. The abdominal wall is closed as in those cases in which a Mickulicz drain is employed.

The following is a brief summary of thirty-two cases treated at the Cook County Hospital, Chicago:

1. The youngest patient was 18; one was 19 years. The oldest 42, the next oldest 40. Eleven more were between 30 and 40, 15 between 20 and 30.

2. Only in three cases was a history of previous sterility present. Sixteen had had one or more miscarriages. One patient was the mother of seven children. Another of eight; two had four children each.

3. The ordinary sign of intra-uterine pregnancy (tender breasts, enlarged uterus, softened cervix) were noted in about one-half of the cases.

4. Pain in the lower abdominal region, menstrual irregularities, continuous vaginal hemorrhage, and a palpable mass in one or the other, or even in both fornices, was present, respectively, in thirty, twenty-eight and thirty-one cases. Those three symptoms are placed under a special heading, because I believe that their presence is, at the very least, highly suggestive of ectopic gestation:

(a) A pelvic pain usually referred to one ovarian region. (The best clinicians and observers agree that tenderness or palpation of the mass adjacent to the uterus is of great diagnostic value.)

(b) Menstrual irregularities, amenorrhea, etc.

(c) A mass palpable vaginally or abdomino-vaginally to one side of uterus connected with or distinct from the latter organ.

(d) In rupture, signs of internal hemorrhage, varying from increasing pallor and fainting spells, up to total collapse.

5. Conception occurred in the right Fallopian tube seventeen times, fourteen in the left, and in one case the side was not determined.

6. Thirty cases were treated by the abdominal route, one by the vaginal route and one by the combined route. The latter patient died. Drainage was used in nine cases. Alarming capillary oozing necessitated the use of the drain in nearly all of these cases.

7. Two deaths occurred in this series, both due to a general peritonitis. Numerous adhesions had to be torn in one case and the other was a case of advanced pregnancy. All the other cases made rapid and uncomplicated recoveries.

